# On the Teaching of Operative Surgery

**Published:** 1990-12

**Authors:** Ronald Belsey

**Affiliations:** Honorary Consultant Thoracic Surgeon, Bristol Royal Infirmary and Frenchay Hospital


### On the Teaching of Operative Surgery

**Ronald Belsey MS FRCS**
Honorary Consultant Thoracic Surgeon, Bristol Royal Infirmary and Frenchay Hospital
Address to the Faculty of Medicine at The University of Palermo on the occasion of receiving an honorary degree.

The prelude to a training in operative surgery is intensive exposure to many disciplines. A working knowledge of anatomy, and especially visceral anatomy as opposed to the skeletal and neuro-muscular anatomy of the text books, is essential. This knowledge will receive further refinement later in the operating room, the final school of practical anatomy.

An awareness of the pathology of the lesion, the possible local extent, common complications, and associated conditions possibly calling for synchronous correction, will promote a clearer understanding of the proceedures at which he is about to assist. "As is your pathology, so is your practice" (Sir William Osler). He will have been involved in the diagnostic investigations where physiology, normal and abnormal, play a dominant role. Diagnosis precedes treatment, and armed with a reasonably accurate diagnosis he can approach the question of the indications for, and contra-indications to, surgical intervention on the assumption that he, the trainee, might be in full charge of the case. He will have asked himself five questions. 1: Is an operation necessary? 2: Is it kind? 3: Am I competent to cope with the problem? 4: Can I reasonably guarantee a satisfactory result? 5: Would I personally submit to the procedure in similar circumstances? The answer to this last question will be the real test of his moral sincerity. If he can answer these five questions with conviction he is ready to enter the operating room and receive his introduction to operative technique. He will have listened to his instructor discuss with the anaesthetist any relevant problems. Chronic aspiration pneumonitis may call for more aggressive airway toilet. Will he need complete muscular relaxation? The probable duration of the procedure and the possible blood loss. Anaesthesia alone can incur significant risks and it is the ultimate responsibility of the surgeon to minimise these risks.

It may be possible to teach the principles of a surgical procedure by a written or illustrated description but not the subtleties of operative technique. Only the time honoured "apprentice" principle and repeated demonstration can achieve this aim. The trainee is now literally in the hands of his instructor whose first responsibility is to demonstrate the basic principles of good operative technique: decision, deliberate planned manoeuvres, gentleness, clean cutting and no rough handling of tissues. Excessive handling of structures stems from an ignorance of the anatomy. Blunt dissection of tissue planes may be permissable with sensitivity and restraint. There must be no haste but no purposeless time wasting activity. Speed in operating should be an achievement and not an objective. The commonest time waster is indecision and lack of planning. Gentleness is perfectly compatible with decisive non-repetitive manoeuvres. It has been claimed that there is no more dangerous person than a surgeon with time on his hands. Of supreme importance is the demonstration of that fundamental virtue of the good surgeon: equanimity, and the ability to deal calmly with any untoward event during the procedure.

Adequate exposure is essential. If the trainee cannot see every manoeuvre, he cannot learn. Each step should be calmly explained as it is performed till the assistant is fully acquainted with the proceedure. "Keyhole surgery" a regretable source of personal pride to some surgeons, is unacceptable in any teaching program. Long incisions, if anatomically planned, heal just as rapidly as short incisions and with less postoperative pain resulting from the over aggressive use of mechanical retractors.

Equally important is the attitude of the instructor to his assistants. Unnecessary comments on clumsiness, flashes of irritation and bad temper, offensive comments to the scrub nurse, are the antithesis of equanimity. Such behaviour in the operating room may appeal to the "prima donna" ethos of some surgeons but will certainly inhibit the assistant trying to be helpful and to learn.

At this stage of his training the student must be taught how to watch an operation profitably. It is a basic principle that nobody can absorb the subtleties of any procedure till he has attempted it personally. A principle I have always adopted is to allow an assistant quite early on in his training to start an operation without my presence in the room, but on the end of a telephone in the hospital. He spends twenty minutes towelling up the patient dismayed by the sudden realisation that although he has assisted on many occasions he has seen nothing. He is rescued from his dilemma before he can commit any irreparable havoc but from that moment he has learnt the most important lesson of his career: how to watch and assimilate.

Toward the finale of his training in operative technique he will hopefully be competent to attempt the simpler procedures. Whether his instructor should assist him in his initial attempt is debatable. I speak from personal experience. It is probably the last case on the list and he may be embarrassed by the conviction that his senior is anxious to get home, or out to the golf course. He may be impelled to make haste, an unforgivable mistake at this stage. Far better that he be allowed to proceed at his own pace with the reassurance that help is at hand, on the end of a phone, if necessary. When he has learnt to watch while assisting at more complex procedures his progress will be rapid and assisting can become meaningful. When finally launched into surgical practice he will continue to benefit from an occasional refresher course in the hands of his instructor. Experience is the name we all give to our mistakes, but he will learn more from these than from his more successful adventures.

A word of warning. A trainee may become so confident in his ability that the first time he operates alone he may aspire to improve on the surgical principles and technique of his mentor. With further experience, and a little humility, he will hopefully learn that such premature modifications and improvements may prove counterproductive.

Surgical technique does not end with the insertion of the final stitch of the wound closure. Every surgical operation is an experiment in pathology and accurately and promptly recorded details of the procedures, and the outcome, are the surgical history of tomorrow. Any report delayed for 24 hours, or longer, or dictated by other than the operating surgeon, is useless.

Postoperative care is the final technical responsibility of the operating surgeon. The anaesthetist may be temporarily responsible for the patient in the recovery room till he is extubated. But it is the surgeon who should review the immediate postoperative chest x-ray, the fluid balance of the patient, and decide whether the patient should be inflicted with the chaos of the ICU or transferred back to the relative calm of his own ward and the nursing staff with whom he is already acquainted.

*Continued on page 122*

On the Teaching of Operative Surgery *continued from page 102*

The follow-up philosophy assumes that every surgical operation lasts for the duration of the patient's, and the surgeon's, life. Operative technique is meaningless unless correlated with the long term result. Eventually the young surgeon will be incited to publish for a variety of motives: prestige, vanity, the "numbers game", economic advancement, or peer pressure in an academic environment. The sole justification for publication is a statistically significant series of cases, followed with strictly objective assessment for a minimum period of five years, or indefinitely beyond that time, and the conviction that he has a message that will contribute to the advancement of surgery rather than to his personal practice. For role models in his literary style he can do no better than turn to the masters of the 18th and 19th centuries, when surgeons had time to write. He should endeavour to cultivate a style that is concise, informative, objective, and moreover readable. He should ever remain mindful of the aphorism that only the greatest intellects can afford to be brief.

In conclusion the potential value of video recordings in the teaching of operative surgery must be reviewed critically. For obvious reasons only brief episodes, that appeal to the surgeon, are recorded. The technically difficult and time consuming stages of the procedure are deleted. The edited version presents a quick, facile performance that may bear little relationship to reality. A trainee may be tempted to try the procedure, with disastrous results, unless he has assisted in the operating room for the entire duration of the procedure and is fully aware of the technical problems that may be encountered. The only positive message from the video is the demonstration of adequate exposure, dictated by the camera rather than the teaching of operative surgery. Video recordings may be of more value to the fully trained surgeon who has already acquired some practical experience of the procedure and is already aware of the practical difficulties that may be encountered.

